# Factors influencing adolescents' attitudes towards vaccination

**DOI:** 10.1016/j.clinsp.2025.100824

**Published:** 2025-11-12

**Authors:** Pelin Dağ

**Affiliations:** Mersin Şehir Eğitim ve Araştırma Hastanesi, Mersin, Turkey

**Keywords:** Covid-19, Adolescent, Death anxiety, Vaccine, Healthcare worker

## Abstract

**Objectives:**

This study aimed to determine the predictors of death anxiety and vaccination attitudes among adolescents during the COVID-19 pandemic and to analyze the association between these variables.

**Materials and methods:**

This cross-sectional, analytical, and descriptive study included 270 adolescents. Data were collected using the Revised Death Anxiety Scale, and a researcher-designed questionnaire assessing sociodemographic characteristics and attitudes toward COVID-19 vaccination.

**Results:**

Vaccination rates varied significantly based on maternal education, with 90 % of unvaccinated adolescents having mothers with undergraduate degrees. Adolescents whose mothers were unemployed had the highest unvaccinated rate (78 %). 13 % of adolescents with health professional parents were unvaccinated. Vaccinated adolescents had higher testing rates for suspected COVID-19 (*p* = 0.016). Vaccination was significantly associated with the death of a relative from COVID-19 (*p* = 0.048), and fear of dying from COVID-19 was higher in vaccinated adolescents (*p* = 0.003). A strong correlation was also found between the fear of losing a loved one and vaccination status (*p* = 0.001).

**Conclusions:**

The study suggests that adolescents' anxiety regarding COVID-19 is strongly associated by the perceived risks of transmission and severity. Consequently, addressing these anxieties and providing clear information about vaccination as a preventive measure may enhance vaccination rates. Additionally, parental education and occupation should be considered as key factors in shaping adolescents' health behaviors and decisions.

## Highlights

• A young person's vaccination decision is influenced by multiple factors.
• Parents' education and occupation significantly affect children's vaccination decisions.
• Adolescents tested for suspected COVID-19 had higher vaccination rates.

## Introduction

For almost two years, the world has faced extraordinary times due to the COVID-19 pandemic. More than a million people have died, and many more have fallen ill, with the number increasing by the day. In addition, the pandemic has led to increased psychological distress due to fear of contracting the disease, the possibility of losing a loved one, quarantine measures, and uncertainty, as well as social, political, and economic issues.^1^^,^^2^ Adolescents were also particularly vulnerable to adverse mental health outcomes due to their developmental and emotional needs, and previous research showed an increase in mental health problems among adolescents during the pandemic.^3^

The COVID-19 pandemic not only had a social, political, and economic impact on society but also an emotional impact on a significant portion of the population.^4^ Psychological stress due to the outbreak, fear of contracting the disease, quarantine practices, and the uncertainty surrounding the pandemic led to a tsunami of mental health problems, including increased levels of depression and anxiety, post-traumatic stress disorder, and eating disorders.^5^ Factors such as limited peer interaction due to online education, increased feelings of isolation and loneliness, changes in daily routines, heightened family conflicts, and economic challenges have all contributed to the worsening mental health of adolescents, who are already emotionally vulnerable.^6^^,^^7^

Throughout history, infectious diseases have caused varying degrees of death and have occasionally caused major epidemics of global concern.^8^ The fear of death, a common existential concern, has gained particular attention in the context of the COVID-19 pandemic. Death anxiety, a natural emotional response to the awareness of mortality, has been exacerbated by the pandemic's high mortality rate.^9^ This phenomenon is particularly pertinent to adolescents, who, while recognizing the inevitability of death, often believe in their personal invulnerability. The pandemic’s impact on death anxiety in adolescents, and its potential association with vaccine acceptance, remains largely unexplored. Research indicates that increased mortality risk typically heightens death anxiety, yet its relationship with vaccination attitudes in adolescents has not been investigated.

During the COVID-19 pandemic, public health safety measures became crucial. Wearing masks, maintaining physical distance, and hand hygiene have been key to reducing transmission. However, these measures alone were insufficient to eliminate the virus, and adherence to them over the long term became increasingly difficult for the public.^10^ Vaccination, therefore, emerged as a vital solution to protect individuals and communities, contributing to herd immunity. While some people see vaccines as a beacon of hope to end the pandemic, others appear to have concerns about getting vaccinated. Factors such as licensing before Phase III trials have been completed, insufficient information about long-term side effects, or concerns about vaccine shortages may cause some individuals to be apprehensive about vaccination.

The COVID-19 vaccines prepare the immune system to recognize the spike protein of the virus, reducing the severity of illness, hospitalizations, and mortality. However, vaccine acceptance is not universal. Factors such as public trust in vaccines, socio-economic disparities, and psychological factors play a major role in vaccination uptake. Specifically, perceived risk and the belief in the benefits and safety of vaccines play a critical role in determining individuals’ willingness to vaccinate. Psychological factors, such as fear of death, have been shown to influence health behaviors, including vaccination.

In addition to psychological variables, family dynamics — particularly parental effect — may play a crucial role in shaping adolescents’ health-related behaviors. Parental education level, occupation, and attitudes toward health risks may function as indirect cues to action. According to the Health Belief Model (HBM), individuals’ likelihood of engaging in a preventive health behavior, such as vaccination, is shaped by their perceived susceptibility to a disease, perceived severity, perceived benefits, perceived barriers, and cues to action. Similarly, the Theory of Planned Behavior (TPB) suggests that intentions to perform health behaviors are influenced by attitudes, perceived social norms (e.g., parental expectations), and perceived behavioral control. Therefore, integrating these theoretical frameworks may provide a more comprehensive understanding of how parental and psychological factors are associated with adolescent vaccination attitudes.^11^

This study was prompted by the urgent need to explore the psychological burden of the COVID-19 pandemic on adolescents — particularly death anxiety — at a time when the virus continues to claim over 200 lives daily, yet remains largely unexamined in this vulnerable population.

This study was designed to explore the relationship between death anxiety and vaccination attitudes in adolescents during the COVID-19 pandemic. By focusing on how parental factors and psychological distress are associated with adolescents’ health behaviors, we aim to fill an important gap in the existing literature.

## Materials and Methods

The study was designed as a cross-sectional, analytical, and descriptive study to determine the level of death anxiety and the factors that associated with death anxiety in healthy adolescents, in alignment with the STROBE guidelines. This design was chosen due to its suitability for capturing a snapshot of adolescent anxiety levels and identifying associated factors within a limited time frame. RDAS (Revised Death Anxiety Scale) consists of a total of 25 items, and based on a literature review, it was determined that including 250 adolescents — 10 times the number of questions — would provide a robust sample size for reliable analysis.

### Selection of healthy adolescents

The questionnaire was administered to adolescents aged 12–18 years who attended the Child and Adolescent Psychiatry Outpatient Clinic, Mersin City Training and Research Hospital, Mersin, Türkiye for routine consultations between November 15, 2021, and December 15, 2021. Adolescents with fully accessible parental information were included in the study. Adolescents with psychiatric disorders or conditions that could impair functioning were excluded to avoid confounding factors. Parents were initially contacted to inform them about the study and obtain their consent.

Exclusion criteria also included adolescents with a history of chronic illness or the loss of a parent unrelated to COVID-19, as these factors might affect their fear of death and confound the study results. After providing an explanation of the study, literate adolescents and their parents who met the inclusion criteria were asked to provide written informed consent. Only those who gave informed consent were included in the study.

### Data collection

A questionnaire was used to collect both socio-demographic data and data from the Revised Death Anxiety Scale (RDAS). Two forms were used to gather data from all adolescents included in the study.

### Sociodemographic questionnaire

The socio-demographic questionnaire was designed by the investigators after a thorough review of the literature to identify relevant factors affecting adolescents' death anxiety, particularly during the COVID-19 pandemic. The first section included questions on the adolescent's age, gender, school grade, number of siblings, family type, district of residence, family income, and the parents' age, education level, and occupation.

The second section gathered information regarding the adolescent’s awareness of the current COVID-19 situation. Adolescents were asked whether they felt sufficiently informed about COVID-19 and the role of social media. They were also inquired whether they or their family members had been diagnosed with COVID-19 and whether any family members had died from the virus. Additional questions assessed the impact of the pandemic on the adolescents' socialization, daily activities, school success, and psychological well-being. Finally, questions regarding their vaccination status, their views on vaccine effectiveness, parental influence on vaccine decisions, and their level of fear regarding contracting COVID-19 or dying were included.

### Revised death anxiety scale (RDAS)

The Revised Death Anxiety Scale (RDAS), developed by Thorson and Powell, was adapted to Turkish by Yıldız and Karaca (2001). The scale’s Cronbach's alpha coefficient was 0.84, with a reliability coefficient of 0.73, calculated using the halving technique.^12^ The reliability and validity studies for adolescents aged 12–18 years were conducted by Ak and Conk, with a reliability coefficient of 0.91.^13^

### Study design and informed consent

The aim of the study was explained to all participants and their families. They were informed that the study posed no risk to either the family or the adolescent, that no interventions could harm the individual, and that all personal information would remain confidential. Written voluntary informed consent was obtained from both the parents and the adolescent participants. Following the consent process, face-to-face interviews were conducted in a non-parental setting with those adolescents who agreed to participate.

A preliminary study was carried out to ensure the clarity of the questions. This pre-test involved 10 adolescents who shared characteristics similar to those of the study participants. After reviewing the responses and finding no need for adjustments, the final version of the questionnaire was used for data collection.

### Ethics committee approval

The study was reviewed and approved by the Ethics Committee for Non-Interventional Studies at the Faculty of Medicine, Toros University. The approval number granted was 78017789/050.01.04/2064146.

## Results

A total of 300 adolescents were invited to complete the Revised Death Anxiety Scale for Adolescents and the socio-demographic data form, following parental consent. Participants aged 12–18 years who met the inclusion criteria and visited the clinic during the study period were included. Adolescents who responded with "I do not know" in the socio-demographic form were excluded. Ultimately, 270 adolescents who completed both forms were included in the analysis.

Among the participants, 150 (55.5 %) were girls, and 120 (44.5 %) were boys. The average age of the adolescents was 16.1 years (range: 14–18).

The mean scores on the Revised Death Anxiety Scale (RDAS) were statistically analyzed, and the results were examined in relation to socio-demographic data to identify factors associating these scores.

A one-way analysis of variance (ANOVA) was conducted between the mean RDAS scores and age groups. No statistically significant difference was found (*p* = 0.154).

An independent samples *t*-test was used to assess the difference in mean RDAS scores between boys and girls. Although girls had higher RDAS scores (50.34 ± 11.24) compared to boys (47.7 ± 10.59), the difference was not statistically significant (*p* = 0.055).

Family characteristics were also explored as potential factors affecting RDAS scores. Regarding family income, no significant differences were observed in RDAS scores (*p* > 0.05). Maternal occupation showed a statistically significant relationship with RDAS scores (*p* = 0.02), whereas no significant relationship was found with maternal education (*p* > 0.05). Adolescents whose mothers were unemployed had the highest RDAS scores (51.4), while those with mothers who were health professionals (44.07) and self-employed had the lowest (43.75). No statistically significant difference was observed for fathers' education or occupation (*p* > 0.05).

Fifty-eight and a half percent of the adolescents were tested for suspected Covid-19. However, there was no significant correlation between testing and RDAS scores (*p* = 0.297). Of the adolescents, 15.6 % had been diagnosed with Covid-19, and no statistically significant association with RDAS scores was found (*p* = 0.417). None of the adolescents were hospitalized due to Covid-19 infection. Forty-eight percent of adolescents expressed some fear of contracting Covid-19, but this did not correlate significantly with RDAS scores (*p* = 0.262).

Twenty-six percent of adolescents reported being very afraid of death, while 44 % were somewhat afraid, and 30 % were not afraid. A significantly higher RDAS score was found among those who were very afraid of dying from Covid-19 (*p* = 0.014).

Eighty-one percent of adolescents had a relative diagnosed with Covid-19, and although this raised the RDAS score from 46.3 to 49.53, the association was not statistically significant (*p* = 0.059). Thirty-five point two percent of adolescents had lost a relative to Covid-19, but the correlation with RDAS scores was not statistically significant (*p* = 0.43). Sixty-eight point nine percent of adolescents reported being very afraid of losing a loved one, but the relationship between this fear and RDAS scores was not statistically significant (*p* = 0.156) (Table 1).

**Table 1:** Relationship between sociodemographical variables and RDAS scores.

		n	mean	St dev	Min p	Max p	p
Gender	Girl	150	47,77	10,59	20,00	75,00	0055
	Boy	120	50,34	11,24	25,00	84,00	
Income	High	44	48,84	11,77	20,00	76,00	0224
	Middle	218	48,69	10,43	23,00	75,00	
	Low	8	55,50	17,91	38,00	84,00	
Mother occupation	Unemployed	162	48,86	11,61	20,00	84,00	0,02*
	Civil Servant	69	51,67	9,18	32,00	75,00	
	Free-lancer	16	43,75	12,20	28,00	66,00	
	Healthcare pr	14	44,07	7,32	35,00	56,00	
	Retired	9	45,56	7,58	35,00	60,00	
Mother Education	Illiterate	4	47,00	14,28	38,00	68,00	0933
	Primary edu	63	48,30	12,25	23,00	84,00	
	High-school	104	49,01	10,89	20,00	72,00	
	University	99	49,28	10,10	25,00	75,00	
Father Education	Illiterate	5	43,20	11,78	35,00	64,00	0537
	Primary edu	42	48,64	11,83	30,00	84,00	
	High-school	106	49,75	11,37	20,00	74,00	
	University	117	48,50	10,20	26,00	76,00	
Father occupation	Unemployed	6	44,00	14,79	35,00	74,00	0096
	Civil Servant	106	49,58	10,54	25,00	83,00	
	Free-lancer	121	49,89	11,43	20,00	84,00	
	Healthcare pr	8	42,88	7,49	35,00	56,00	
	Retired	26	45,27	9,40	23,00	59,00	
Covid-19 testing	Yes	158	48,33	10,90	20,00	83,00	0297
	No	112	49,74	10,98	28,00	84,00	
Diagnosed covid-19	Yes	42	47,69	11,56	25,00	83,00	0431
	No	228	49,14	10,83	20,00	84,00	
Relatives diagnosed as covid-19	Yes	218	49,53	11,06	23,00	84,00	0059
	No	52	46,35	10,13	20,00	69,00	
Decedent relative due to Covid-19	Yes	95	49,62	11,26	23,00	83,00	0435
	No	175	48,53	10,78	20,00	84,00	
Thought on Vaccine efficacy	Sufficient	211	49,49	10,77	23,00	83,00	0259
	Insufficient	20	46,40	9,46	29,00	74,00	
	Indecisive	39	47,10	12,35	20,00	84,00	
Vaccination	Yes	229	49,47	11,22	20,00	84,00	0049*
	No	41	45,83	8,69	29,00	74,00	
Fear of getting Covid-19	Unfeared	141	49,84	10,05	26,00	84,00	0262
	Little scared	108	47,58	11,50	20,00	76,00	
	Very scared	21	49,52	13,35	29,00	83,00	
Fear of Death	Unfeared	80	47,74	10,15	29,00	84,00	0014*
	Little scared	121	47,81	10,12	20,00	72,00	
	Very scared	69	52,22	12,57	23,00	83,00	
Fear of losing beloved one	Unfeared	16	46,38	12,62	29,00	74,00	0156
	Little scared	68	47,18	9,70	28,00	84,00	
	Very scared	186	49,77	11,16	20,00	83,00	

Regarding the Covid-19 vaccine, 85 % of adolescents were vaccinated, and those who were vaccinated had a higher mean RDAS score (49.47) compared to the unvaccinated group (45.83). A significant correlation was found between vaccination and RDAS scores (*p* = 0.049).

No significant differences were found between gender and age concerning anti-vaccination attitudes (*p* = 0.79 and *p* = 0.58, respectively). However, maternal education level was significantly associated with vaccination status (*p* = 0.001). Only 4 % of unvaccinated adolescents had mothers with a graduate degree or higher, whereas 90 % of unvaccinated adolescents had mothers with an undergraduate degree. Similarly, maternal occupation was significantly related to vaccination (*p* = 0.006), with unemployed mothers having the highest proportion of unvaccinated adolescents (78 %). Significant differences were also found between fathers' education and occupation, and vaccination rates (*p* = 0.001 for both).

Vaccinated adolescents had a higher rate of Covid-19 testing (*p* = 0.016). However, no significant correlation was found between Covid-19 diagnosis and vaccination status (*p* = 0.448). A significant association was found between having a vaccinated relative and Covid-19 diagnosis (*p* = 0.033), with higher rates of Covid-19 infection or death in unvaccinated adolescents. Similarly, the death of a relative due to Covid-19 was significantly associated with vaccination status (*p* = 0.048).

There was no significant correlation between fear of contracting Covid-19 and vaccination status (*p* = 0.086), but fear of dying from Covid-19 was significantly associated with vaccination (*p* = 0.003). Among those not afraid of dying from Covid-19, the rate of non-vaccination was significantly higher (26 %). Additionally, fear of losing a loved one was higher among unvaccinated adolescents, and a significant relationship was found between losing a loved one due to Covid-19 and vaccination status (*p* = 0.001). Eight out of 16 adolescents (50 %) who were not afraid of losing a loved one were unvaccinated (Table 2).

**Table 2:** Relationship between vaccination and sociodemographical variables.

			Vaccinated	Not vaccinated	Total	p
Gender	Girl	Count	128_a_	22_a_	150	0791
		Rate	55,9 %	53,7 %	55,6 %	
	Boy	Count	101_a_	19_a_	120	
		Rate	44,1 %	46,3 %	44,4 %	
Income	High	Count	37_a_	7_a_	44	0477
		Rate	16,2 %	17,1 %	16,3 %	
	Middle	Count	184_a_	34_a_	218	
		Rate	80,3 %	82,9 %	80,7 %	
	Low	Count	8_a_	0_a_	8	
		Rate	3,5 %	0,0 %	3,0 %	
Maternal Occupation	Unemployed	Count	130_a_	32_b_	162	0006*
		Rate	56,8 %	78,0 %	60,0 %	
	Civil Servant	Count	68_a_	1_b_	69	
		Rate	29,7 %	2,4 %	25,6 %	
	Free-lancer	Count	12_a_	4_a_	16	
		Rate	5,2 %	9,8 %	5,9 %	
	Healthcare pr	Count	12_a_	2_a_	14	
		Rate	5,2 %	4,9 %	5,2 %	
	Retired	Count	7_a_	2_a_	9	
		Rate	3,1 %	4,9 %	3,3 %	
Maternal Education	Illiterate	Count	3_a_	1_a_	4	0001*
		Rate	1,3 %	2,4 %	1,5 %	
	Primary edu	Count	48_a_	15_b_	63	
		Rate	21,0 %	36,6 %	23,3 %	
	High-school	Count	83_a_	21_a_	104	
		Rate	36,2 %	51,2 %	38,5 %	
	University	Count	95_a_	4_b_	99	
		Rate	41,5 %	9,8 %	36,7 %	
Father Education	Illiterate	Count	1_a_	4_b_	5	<0001*
		Rate	,4 %	9,8 %	1,9 %	
	Primary edu	Count	27_a_	15_b_	42	
		Rate	11,8 %	36,6 %	15,6 %	
	High-school	Count	91_a_	15_a_	106	
		Rate	39,7 %	36,6 %	39,3 %	
	University	Count	110_a_	7_b_	117	
		Rate	48,0 %	17,1 %	43,3 %	
Father Occupation	Unemployed	Count	1_a_	5_b_	6	<0001*
		Rate	,4 %	12,2 %	2,2 %	
	Civil Servant	Count	98_a_	8_b_	106	
		Rate	43,4 %	19,5 %	39,7 %	
	Free-lancer	Count	102_a_	19_a_	121	
		Rate	45,1 %	46,3 %	45,3 %	
	Healthcare pr	Count	7_a_	1_a_	8	
		Rate	3,1 %	2,4 %	3,0 %	
	Retired	Count	18_a_	8_b_	26	
		Rate	8,0 %	19,5 %	9,7 %	
Covid-19 testing	Yes	Count	141_a_	17_b_	158	0016*
		Rate	61,6 %	41,5 %	58,5 %	
	No	Count	88_a_	24_b_	112	
		Rate	38,4 %	58,5 %	41,5 %	
Diagnosed covid-19	Yes	Count	34_a_	8_a_	42	0448
		Rate	14,8 %	19,5 %	15,6 %	
	No	Count	195_a_	33_a_	228	
		Rate	85,2 %	80,5 %	84,4 %	
Relatives diagnosed as covid-19	Yes	Count	180_a_	38_b_	218	0033*
		Rate	78,6 %	92,7 %	80,7 %	
	No	Count	49_a_	3_b_	52	
		Rate	21,4 %	7,3 %	19,3 %	
Decedent relative due to Covid-19	Yes	Count	75_a_	20_b_	95	0048*
		Rate	32,8 %	48,8 %	35,2 %	
	No	Count	154_a_	21_b_	175	
		Rate	67,2 %	51,2 %	64,8 %	
Fear of getting Covid-19	Unfeared	Count	114_a_	27_a_	141	0086
		Rate	49,8 %	65,9 %	52,2 %	
	Little scared	Count	98_a_	10_b_	108	
		Rate	42,8 %	24,4 %	40,0 %	
	Very scared	Count	17_a_	4_a_	21	
		Rate	7,4 %	9,8 %	7,8 %	
Fear of Death	Unfeared	Count	59_a_	21_b_	80	0003*
		Rate	25,8 %	51,2 %	29,6 %	
	Little scared	Count	110_a_	11_b_	121	
		Rate	48,0 %	26,8 %	44,8 %	
	Very scared	Count	60_a_	9_a_	69	
		Rate	26,2 %	22,0 %	25,6 %	
Fear of losing beloved one	Unfeared	Count	8_a_	8_b_	16	<0001*
		Rate	3,5 %	19,5 %	5,9 %	
	Little scared	Count	59_a_	9_a_	68	
		Rate	25,8 %	22,0 %	25,2 %	
	Very scared	Count	162_a_	24_a_	186	
		Rate	70,7 %	58,5 %	68,9 %	

### Multivariate logistic regression analysis

**Fig. 1:**
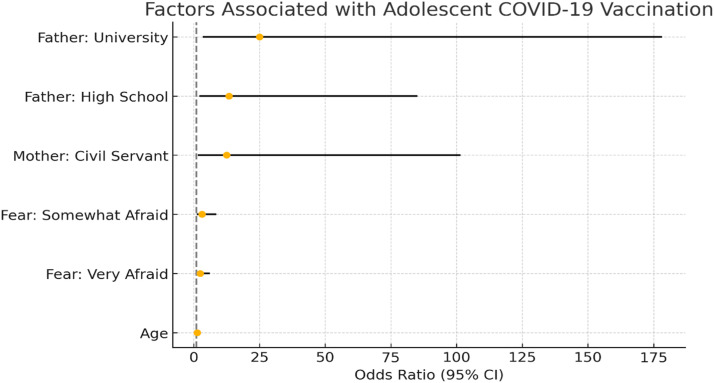
**Odds Ratios for Predictors of Adolescent COVID-19 Vaccination.** This figure displays the odds ratios and 95 % confidence intervals for key predictors of COVID-19 vaccination among adolescents. The vertical dashed line represents the null effect (OR = 1). Values greater than 1 indicate higher odds of vaccination associated with the variable.

Multivariate logistic regression analysis identified several significant predictors of adolescents’ COVID-19 vaccination status. Adolescents whose fathers had completed high school or university were substantially more likely to be vaccinated, with odds ratios of approximately 13.4 and 25.0, respectively. Maternal employment as a civil servant was also strongly associated with increased vaccination likelihood (OR ≈ 12.5, 95 % CI: 1.53–101.5). Additionally, fear of dying from COVID-19 played a notable role; those who reported being somewhat afraid had over three times the odds of being vaccinated (OR ≈ 3.1), while those who were very afraid also showed increased likelihood (OR ≈ 2.45), though with a wider confidence interval. Age was positively associated with vaccination (OR ≈ 1.31), suggesting older adolescents were more likely to be vaccinated. In contrast, overall death anxiety scores (RDAS) were not significantly associated with vaccination status (OR ≈ 1.02). These findings align with theoretical frameworks emphasizing perceived threat and parental role as key drivers of health behavior in adolescents (Fig. 1).

## Discussion

The findings of this study offer a multidimensional view of adolescents’ COVID-19 vaccination attitudes by considering both psychological responses and family-related characteristics. The results particularly highlight the roles of death-related fear and parental background in shaping adolescents’ decisions regarding vaccination.

The effect of gender on people's attitudes has been recognized and studied by psychologists and sociologists. In a study of death anxiety using the Templer Death Anxiety Scale, women were found to have significantly higher levels of death anxiety than men.^14^ This was thought to be due to the fact that girls are more prone to reactions of fear and anxiety and do not hide their feelings about the subject. However, it was found that the mean scores were higher for girls than for boys, although the difference was not statistically significant.

One of the strengths of this study is the evaluation of factors other than fear of death that affect adolescents‘ views on Covid-19 vaccine and adolescents’ attitudes towards vaccination. In this study, gender, age, and family income did not make a difference in whether or not to vaccinate. Previous studies have shown that women in Turkey have a more ambivalent attitude towards vaccination than men.^14^ Although there were no studies in adolescents, no gender difference in vaccine uncertainty was found in this study.

Family characteristics were examined as factors affecting RDAS outcomes in adolescents. Although the pandemic affected families in many ways, income level showed no statistically significant relationship with RDAS scores. Similarly, the father's education and occupation were not associated with RDAS. However, the mother's occupation was significantly related: adolescents with civil servant mothers had the highest RDAS scores, while those with health worker or self-employed mothers had lower scores. Maternal education level did not show a significant effect.

Beyond its association with death anxiety, family structure also appeared to play a meaningful role in adolescents’ vaccination behaviors. Vaccinated adolescents more often had university graduate parents, particularly civil servants, while non-vaccinated adolescents had higher rates of non-working parents. The rates of health worker parents were similar across both groups. This pattern may be interpreted through the Theory of Planned Behavior, which emphasizes the shaping role of parental norms and perceived control.

Previous studies in adults have found higher vaccine acceptance among the elderly, chronically ill, and highly educated, but lower acceptance among those with COVID-19 history. Özceylan et al. found high vaccine ambivalence among people with low education or vaccine literacy.^15^ Although similar studies are lacking for adolescents, this study showed that parental education influenced vaccination. Most unvaccinated adolescents had mothers with only bachelor’s degrees, and housewife mothers were associated with the highest non-vaccination rate.

In addition to maternal factors, the father’s education and occupation were also relevant. The lowest non-vaccination rates were among adolescents whose fathers were civil servants or health professionals. Multivariate logistic regression identified paternal education, maternal occupation, age, and fear of death as significant predictors of vaccination. University-educated fathers increased the odds of vaccination nearly sevenfold, and civil servant mothers more than thirteenfold.

These findings reflect the role of family structure and perceived threat, consistent with behavioral theories. The association between death-related fear and vaccine uptake suggests that public health messaging should acknowledge fear while promoting protection. Notably, maternal education and household income were not significant predictors in the multivariate model, possibly due to stronger paternal modeling or occupational influence.

Some occupational groups face death more frequently in their professional roles. Health professionals, for example, are often exposed to life-threatening scenarios, which may affect how death is perceived by their children. High-risk professions like police and firefighters show lower death anxiety, unlike lower-risk occupations. There is little data on adolescents in these environments. Self-employed parents may have faced higher economic pressure, potentially influencing adolescent anxiety. Additionally, the mother’s occupation may have significantly shaped the household routine during lockdowns, influencing adolescents' emotional states.

Parents who are health professionals are addressed as a separate topic here. Healthcare workers and some professional groups continued to work during the Covid-19 pandemic. However, some groups such as housewives, students, or elderly people were isolated at home for a long time. Having a mother who works in health services may have been protective for the adolescent in terms of accessing health services or creating the idea that the mother has sufficient knowledge about Covid-19.

In addition to parental occupational roles, adolescents’ personal experiences with the disease were also considered in relation to their psychological responses. Suspicion or diagnosis of Covid-19 did not significantly affect RDAS scores. Since adolescents often experience asymptomatic infections, none were hospitalized. Testing procedures and isolation created emotional strain, limiting communication with relatives and increasing stress. However, with the rollout of vaccination and increased public knowledge, fear of death may have decreased. Approximately half of the adolescents reported experiencing some level of fear regarding contracting COVID-19; however, this fear did not exhibit a statistically significant correlation with RDAS scores. Consistent with prior literature, the psychological burden of the pandemic has been shown to disproportionately affect younger populations.^16^ Existing evidence also suggests that vaccine hesitancy tends to be higher among individuals who have personally experienced COVID-19 or had infected relatives. In the present study, adolescents who had undergone testing for suspected infection demonstrated significantly higher vaccination rates, yet no meaningful association was found between confirmed COVID-19 diagnosis and vaccination status.

While fear of infection itself was not linked to vaccination behavior, fear of death due to COVID-19 emerged as a significant motivational factor among vaccinated adolescents. This supports the notion that perceived severity, rather than susceptibility alone, may drive vaccine uptake. Furthermore, adolescents who had previously contracted the virus — or believed they had experienced asymptomatic infection — may have perceived themselves as protected, reducing their perceived need for vaccination. This perceived immunity could also explain the observed inverse relationship between prior infection and fear responses. Such patterns suggest a potential desensitization effect among adolescents with direct exposure to the virus.

Despite 80 % of adolescents reporting that a relative had been diagnosed with COVID-19, this exposure did not result in a statistically significant increase in RDAS scores. Likewise, neither the death of a relative due to COVID-19 (reported by 15 % of participants) nor the fear of losing a loved one demonstrated a significant relationship with adolescents’ death anxiety levels. These findings suggest that familial exposure to the disease alone may be insufficient to elevate death-related distress among adolescents, potentially due to emotional desensitization or adaptive coping mechanisms.

Interestingly, while vaccinated adolescents more frequently reported fear of dying and had undergone COVID-19 testing, unvaccinated adolescents were disproportionately represented among those who had experienced illness or death within their family circles. This inverse association may reflect a complex psychological mechanism in which direct exposure to trauma reduces perceived efficacy or trust in preventive measures, such as vaccination. Such mistrust, possibly rooted in healthcare skepticism or fatalistic attitudes, may contribute to vaccine hesitancy—an area warranting further qualitative investigation. Moreover, the absence of fear regarding the potential loss of loved ones was notably more common in the unvaccinated subgroup, further emphasizing the potential role of emotional distancing in shaping vaccine-related decisions.

Although empirical studies focusing specifically on adolescents remain scarce, the present study identified a significant association between vaccination status and familial COVID-19 experiences, particularly the diagnosis or death of a relative. Strikingly, these events were more frequently reported among unvaccinated adolescents. One plausible explanatory factor is the influence of parental education: lower educational attainment may be associated with limited health literacy and suboptimal information processing regarding vaccine safety and efficacy. Prior research supports this hypothesis, highlighting that inadequate health education can undermine adolescents' attitudes toward vaccination and risk perception.^17^

This study is subject to several limitations. First, its cross-sectional design restricts the ability to establish causal relationships; therefore, all observed associations should be interpreted as correlational rather than causal. Second, data collection was limited to a single outpatient clinic in Turkey, which may constrain the generalizability of the findings across diverse cultural and socioeconomic populations. Third, the sample exhibited a slight gender imbalance (55.5 % female), which could influence psychological variables such as death anxiety.

Future research should prioritize multi-center, longitudinal designs to enhance external validity and better assess temporal relationships. Moreover, targeted public health interventions should consider engaging parents — particularly those with lower educational attainment — as parental involvement emerged as a critical factor influencing adolescents’ vaccination behavior. Tailored vaccine education strategies aimed at families with limited health literacy may prove especially effective in addressing vaccine hesitancy among adolescents.

## Conclusions

This study underscores that adolescents' COVID-19 vaccination decisions are significantly shaped by death-related anxieties — particularly concerns about personal mortality and the potential loss of loved ones. Parental education and occupational status also emerged as key contextual factors influencing adolescents’ health-related choices, further emphasizing the role of family structure in vaccination attitudes. These findings are in line with prominent health behavior theories, highlighting the importance of perceived threat and social norms in shaping preventive behaviors.

While these insights offer meaningful contributions to the understanding of adolescent vaccination attitudes, they should be interpreted within the context of the study’s design and setting. Future research employing longitudinal, multi-site approaches is recommended to deepen these findings and support the development of targeted interventions, especially those aimed at addressing health literacy disparities within families.

## Authors’ contributions

Pelin Dağ: Conceptualization; methodology; formal analysis; investigation; resources; data curation; writing-original draft preparation; writing-review & editing, project administration.

## Conflicts of interest

The authors declare no conflicts of interest.

## Data Availability

The datasets generated and/or analyzed during the current study are available from the corresponding author upon reasonable request.
